# Flaming the fight against cancer cells: the role of microRNA-93

**DOI:** 10.1186/s12935-020-01349-x

**Published:** 2020-06-29

**Authors:** Milad Ashrafizadeh, Masoud Najafi, Reza Mohammadinejad, Tahereh Farkhondeh, Saeed Samarghandian

**Affiliations:** 1 Department of Basic Science, Faculty of Veterinary Medicine, University of Tabriz, Tabriz, Iran; 2 Radiology and Nuclear Medicine Department, School of Paramedical Sciences, Kermanshah University of Medical Sciences, Kermanshah, Iran; 3 Pharmaceutics Research Center, Institute of Neuropharmacology, Kerman University of Medical Sciences, Kerman, Iran; 4 Cardiovascular Diseases Research Center, Birjand University of Medical Sciences, Birjand, Iran; 5 Healthy Ageing Research Center, Neyshabur University of Medical Sciences, Neyshabur, Iran

**Keywords:** Cancer therapy, MicroRNA, Oncogenesis, Oncosuppressor, Signaling pathway

## Abstract

There have been attempts to develop novel anti-tumor drugs in cancer therapy. Although satisfying results have been observed at a consequence of application of chemotherapeutic agents, the cancer cells are capable of making resistance into these agents. This has forced scientists into genetic manipulation as genetic alterations are responsible for generation of a high number of cancer cells. MicroRNAs (miRs) are endogenous, short non-coding RNAs that affect target genes at the post-transcriptional level. Increasing evidence reveals the potential role of miRs in regulation of biological processes including angiogenesis, metabolism, cell proliferation, cell division, and cell differentiation. Abnormal expression of miRs is associated with development of a number of pathologic events, particularly cancer. MiR-93 plays a significant role in both physiological and pathological mechanisms. At the present review, we show how this miR dually affects the proliferation and invasion of cancer cells. Besides, we elucidate the oncogenesis or oncosuppressor function of miR-93.

## Introduction

Cancer is defined as a process that cells undergo uncontrolled proliferation due to abnormal alterations in genetic material. It seems that both internal and external factors are involved in generation of cancer [1]. It has been demonstrated that the enhanced proliferation of cancer cells continues in spite of inhibition of external or internal factors [2]. Accumulating data demonstrates that genetic alterations are mainly responsible for the generation of cancer [3]. During cell proliferation, there are certain points that regulate cell division. Induction of cancer results in lack of response to these modulators [4, 5]. This nonstop proliferation produces benign and malignant tumor cells that would compete for nutritional sources with normal cells [6]. The metastasis nature of cancer cells defines them as malignant or benign. Cancer cells are capable of moving to the various organs and tissues of body via lymphatic chain. This mechanism is known as metastasis. The malignant tumor cells are able to significantly invade other tissues and organs while benign tumor cells are restricted to a certain location [7, 8]. In respect to the potential role of genetic alterations in induction of cancer, investigation of genetic material reveals that microRNAs (miRs) play a remarkable role in cancer progression and migration [9–12].

MiRs are short non-coding RNA molecules with the length of 19–24 nucleotide bases capable of gene regulation at the post-transcriptional level [13, 14]. After the discovery of miRs in 1993 in *Caenorhabditis elegans*, much attention was made towards them to identify and determine their location in genome [15–17]. It appears that this focus on miRs emanates from their critical role in regulation of important biological processes including programmed cell death (both apoptosis and autophagy) [18, 19], cell growth and division [20, 21], cell differentiation [22, 23] and metabolism [24]. A growing body of evidence demonstrates that miRs recognize their target genes by binding to the 3′-untranslated region [25–27]. Based on the role of miRs in biological processes, abnormal expression of miRs is associated with development of a number of pathologic events such as cancer [28, 29], cardiovascular diseases [30, 31] and neurological disorders (NDs) [32, 33]. The alteration in expression leads to the application of miRs as biomarkers for identification of various pathological conditions, particularly cancer. For instance, miR-141 is one of the potential miRs involved in suppressing the malignancy and invasion of laryngeal cancer cells that undergo down-regulation in this cancer [34]. MiR-132 is capable of significant reduction in the viability and proliferation of renal carcinoma cells [35]. These studies highlight this fact that identification of miRs and their subsequent targeting is a promising strategy in treatment of cancer as a life-threatening condition [36].

## Biogenesis of microRNAs

It seems that a number of stages should be performed to produce a mature miR from a long primary transcript. First of all, RNA polymerase II/III synthesizes a hairpin structure known as primary-miR (pri-miR) [37]. Next, RNase III family enzymes (Dicer and Drosha) use pri-miR as a substrate to produce precursor-miR (pre-miR) with a length of about 70 nucleotide bases [38]. After the translocation of pre-miR into cytoplasm via Exportin-5 (Exp5), Dicer enzymes lead to the formation of a short mature miR [39]. This mature miR is not active and should follow a variety of other stages to be activated. The entering of mature miR into miR-induced silencing complex (miRISC) by transactivation-responsive RNA-binding protein (TRBP) results in the interaction between DICER and Argonaute (Ago) proteins. Now, the mature miR is active and miRISC complex chooses a single strand of mature miR to bind to the target gene leading to the translation inhibition or degradation [40, 41].

## MicroRNA and cancer development

Cancer progression is caused by several changes in oncogenes and tumor-suppressor genes during several years [42]. Numerous studies have indicated a main role for miRNAs in the cancer pathogenesis [42]. miRNAs can affect cancer progression through regulation of cell growth and apoptosis [42]. The miRs are used as biomarkers for assessing cancer prognosis and drug efficacy [42]. Many miRs act as oncogenes or tumor suppressors in the cancers process [42]. Thus, the increasing knowledge on these miRs helps us in cancer therapy [42].

## Expression alteration of microRNAs in cancer

Overall, miRs are divided into two major categories known as oncosuppressor and oncogenesis miRs. As it is obvious, oncosuppressor miRs are involved in suppressing the migration and invasion of cancer cells, whereas oncogenesis miRs contribute to the progression and proliferation of cancer cells [42]. There is the third type of miRs known as apoptomiR that can regulate the apoptotic cascade in cancer cells [43]. In tumor cells, the expression of these miRs undergoes alterations. It has been demonstrated that tumor cells are able to enhance the expression of oncogenesis miRs and reduce the expression prolife of oncosuppressor miRs to ensure their malignancy and invasion [44–47]. MiR-152 is an oncosuppressor miR that enhances the potential of chemotherapy by sensitizing cancer cells to apoptotic cell death [48]. MiR-205-5p is another oncosuppressor miR involved in inhibition of proliferation of breast cancer cells [49]. It is held that the down-regulation of oncosuppressor miRs occurs during cancer growth and metastasis [50, 51].

## MicroRNA-93: a brief introduction

Accumulating data demonstrates that miR-93 plays a significant role in both physiological and pathological conditions. Several studies have been conducted to show the down-regulation/upregulation of miR-93 in diseases and health. A recent study shed some light on the role of miR-93 in NDs. It is held that miR-93 contributes to the inflammation by affecting the proliferation of microglia and blood levels of miR-93 are considered as reliable indexes for diagnosis and prediction of functional recovery of acute ischemic stroke [52, 53]. The upregulation of miR-93 occurs in cerebral cortex and hippocampus after transient brain injury [54, 55]. These alterations show the potential of miR-93 of being used as a prognosis signature of transient brain injury [56]. Besides, the expression of miR-93 elevates in the postischemic brain [57]. In respect to the involvement of miR-93 in ischemic injury, application of miR-93 inhibitor is associated with attenuation of the injury by enhancing the antioxidant defense system through nuclear factor erythroid 2-related factor 2 (Nrf2) signaling pathway [58]. A growing body of evidence exhibits that miR-93 predisposes to immune system disorders by stimulation of secretion of cytokines, chemokines and growth factors [59, 60]. Furthermore, miR-93 is able to reduce the expression of signal transducer and activator of transcription 3 (STAT3) to suppress neuroinflammation and consequently, NDs [61]. MiR-93 involves in amelioration of cardiovascular diseases. It appears that miR-93 considerably promotes perfusion recovery and angiogenesis resulting in improvement of peripheral arterial diseases [62, 63]. MiR-93 also is capable of targeting macrophages (macrophage M2 polarization) in elevating angiogenesis and arteriogenesis in PAD [64]. Pharmacological and genetic manipulations of miR-93 are of importance in treatment of pathological conditions [65, 66]. Taking everything into account, it seems that miR-93 has an efficient role in preserving health condition and its abnormal expression is associated with pathologic events. In the next sections, we discuss the impact of miR-93 expression in cancer cells.

## MiR-93 in cancer malignancy

### Lung cancer

Lung cancer is one of the most challenging problems worldwide with high morbidity and mortality [67]. In spite of huge advancement in diagnosis and treatment of lung cancer, the patients with this life-threatening disorder still have a low 5-year survival rate [68, 69]. In respect to the involvement of genetic factors in generation of lung cancer, targeting miRs is of importance in its treatment. Circular RNAs (circRNAs) are a kind of transcripts which produce a covalently closed continuous loop [70, 71]. It is held that circRNAs are able to sponge miRs that is due to their miRNA target sites such as Ago2 protein [72–75]. Based on the vital role of circRNAs in regulation of biological processes, they are of interest in treatment of pathological conditions, particularly cancer [52, 76–79]. In the case of lung cancer, circCRI-M1 remarkably diminishes the invasion and malignancy of tumor cells. Due to the Ago2 protein, this circRNA is capable of sponging the miR-93 that in turn, enhances the expression of leukemia inhibitory factor receptor, as a tumor suppressor leading to the reduced metastasis and viability of lung cancer cells [80]. As one of the potential targets of circRNAs, miR-93-5p is affected by circRNF13 in lung cancer cells. CircRNF13 is capable of reducing the proliferation and viability of lung cancer cells by down-regulation of miR-93-5p through interacting with Ago2 [81]. Non-small cell lung cancer (NSCLC) is responsible for more than third-to-fourth of lung cancers [82]. Although much improvement has been made in the diagnosis and treatment of this life-threatening disorder, it is still one of the leading causes of death. MiR-93-5p acts as a biomarker in NSCLC. The expression of this miR suggested to be higher in NSCLC patients compared to the healthy ones. Besides, its high expression is associated with poor prognosis and low survival of patients with NSCLC. This is due to the stimulatory impact of miR-93-5p on the migration, proliferation, and invasion of NSCLC cells [83]. Based on the role of PI3K/Akt pathway in cancer progression, this molecular pathway is considered as a downstream mediator of miR-93. In order to elevate the malignancy and invasion of NSCLC cells, miR-93 downregulates the expression of tumor suppressor genes including LKB1, PTEN and CDKN1A to stimulate PI3K/Akt signaling pathway and subsequently, ensure the migration and proliferation of these tumor cells [84]. Neural precursor cell expressed developmentally downregulated gene 4-like (NEDD4L) is considered as a NEDD4-like E3 ubiquitin ligase [85]. The main function of NEDD4L is the regulation of ion channel receptors and transporters [86, 87]. However, accumulating data demonstrates that NEDD4L is capable of modulation of a number of signaling pathways such as tumor growth factor-β (TGF-β) [88–91]. It is held that NEDD4L undergoes down-regulation in lung cancer [88]. MiR-93 prevents the expression of NEDD4L to stimulate EMT via TGF-β upregulation [92]. Wnt signaling pathway is suggested to be involved in the modulation of cell proliferation and cell differentiation [93, 94]. These important roles have made Wnt signaling pathway as a vital target in cancer therapy [95, 96]. Zinc and ring finger 3 (ZNRF3) is an inhibitor of Wnt signaling pathway [97]. In lung carcinoma, ZNRF3 reduces the expression of Wnt pathway to inhibit the proliferation of tumor cells. Given the role of miR-93 as an oncogenesis miR, this miR inhibits ZNRF3 to ensure the viability of tumor cells [98]. MiR-93 is a biomarker of NSCLC as its expression elevates in the tissues of patients with NSCLC [99].

The disabled homolog 2 (DAB2) is a tumor suppressor gene encoding a mitogen-responsive phosphoprotein. The expression of DAB2 undergoes down-regulation in various cancers and lung cancer is among them [62, 100–104]. Based on the role of miR-93 in enhancing the malignancy of lung cancer cells, this miR downregulates the expression of DAB2 to promote the proliferation and malignancy of these tumor cells [105].

### Esophageal cancer

Esophageal cancer [106] is one of the most malignant cancers and claim the seventh place among other malignant tumors [107]. Both environmental and genetic factors contribute to the progression of EC [108, 109]. It has been demonstrated that miRs can promote the proliferation and viability of EC cells [110]. Hence, manipulation of miRs can reduce the malignancy of EC cells. A synthetic circRNA resistant to the digestion with RNase R, suppressed the expression of miR-93 as an oncogenesis miR in EC and resulted in diminished proliferation and migration, and enhanced apoptosis of tumor cells [111]. PTEN plays a significant role in modulation of cell cycle and proliferation. It seems that PTEN exerts an anti-tumor impact since its expression undergoes down-regulation during cancer progression [112, 113]. Investigation of molecular pathways demonstrates that miR-93-5p enhances the malignancy and proliferation of EC cells by down-regulation of PTEN/PI3K/Akt axis and its downstream targets p21 and cyclin D1 [114]. MiR-93 can be considered as a potential biomarker of EC as its expression enhances in EC cells [115].

### Osteosarcoma

Osteosarcoma (OS) is a malignant tumor among adolescents [116]. The capability of OS cells in metastasis to neighboring cells and tissues leads to the high death and minimal 5-year survival rate among the patients [117, 118]. Long non-coding RNAs (lncRNAs) are another member of non-coding RNAs with the ability of regulating miRs [119, 120]. It seems that the enhanced expression of lncRNA AWPPH is associated with upregulation of miR-93-3p and subsequently, an increase occurs in the viability and proliferation of OS cells [121]. By induction of miR-93-3p/FZD7 axis, lncRNA AWPPH upregulates the expression of Wnt/β-catenin signaling pathway to enhance the migration and invasion of tumor cells [121].

### Cervical cancer

Cervical cancer is at the second place after breast cancer among gynecological malignancies and negatively affects the women’s health [122]. Fortunately, we have witnessed a remarkable decrease in the incidence rate of cervical cancer due to the powerful tools in diagnosis and further therapy of this disorder [123, 124]. B cell translocation gene 3 (BTG3) inhibition is a promising strategy by tumor cells to decrease the efficacy of radiotherapy [125]. Moreover, BTG3 is considered as a tumor suppressor and its inhibition enhances the malignancy of cervical cancer cells [126]. There is a reverse relationship between miR-93 and BTG3 expression in cervical cancer cells. By down-regulation of miR-93-5p, an increase occurs in the expression profile of BTG3 to stimulate apoptosis and reduce proliferation and metastasis [127].

### Brain tumors

It is held that miR-93 can be used as a prognostic signature of primary central nervous system lymphoma (PCNSL). This is due to the impact of this miR on the various genes. It appears that miR-93 is able to affect a number of factors such as programmed cell death 1 ligand 2 (PDCD1LG2) associated with cancer immunotherapy, G-protein coupled receptor 137C (GPR137C) and mitogen-activated protein kinase 2 (MAPK2). Investigation of miR-93 expression prolife reveals that patients with low expression of miR-93 have poor prognosis that is maybe due to its impact on PDCD1LG2. This study demonstrates that miR-93 can be considered as a reliable biomarker for prediction of prognosis of patients with PCNSL [128]. Gliomas are one of the most common malignant tumors of brain with high aggressiveness [129, 130]. The World Health Organization (WHO) has divided gliomas into four grades (I, II, III and IV) and it has been shown that grade IV is more common compared to the other grades [131]. Genetic alterations are partially responsible for the generation and progression of gliomas. LncRNA MEG3 involves in induction of apoptotic cell death in glioma cells and reducing their proliferation and viability by down-regulation of miR-93 [132]. As a downstream target of PTEN, PI3K/Akt signaling pathway is affected by miR-93 to ensure the malignancy of glioma cells. It is held that the expression of miR-93 undergoes upregulation in the tissues of patients with gliomas and is associated with clinicopathologic grade and overall survival of patients. The activated miR-93 inhibits PTEN, PH domain leucine rich repeat protein phosphatases (PHLPP) and forkhead box O3 (FOXO3) by targeting 3^/^-UTR. The inactivation of these pathways leads to the induction of PI3K/Akt that significantly enhances the malignancy of glioma cells [133].

### Prostate cancer

Bioinformatics analysis shows that miR-93-5p functions as an oncogensis miR during prostate cancer progression. Accumulating data demonstrates that suppressing the expression of miR-93-5p is associated with a decrease in proliferation, migration and invasion of prostate cancer cells, while an increase occurs in apoptosis [134]. Disabled homolog 2 (DAB2) participates in modulation of cancer progression by targeting a number of molecular pathways such as Akt and ERK1/2. It has been reported that stimulation of Akt and ERK1/2 remarkably elevates the malignancy and proliferation of prostate cancer cells and exerts anti-apoptotic impact [135–137]. Based on the role of miR-93 in enhancing the malignancy of tumor cells, this miR reduces the expression of DAB2 to upregulate Akt and ERK1/2 signaling pathways [138]. On the other hand, great tea (*Camellia sinensis*) has high anti-tumor activity [139]. It seems that inhibition of miR-93 and simultaneous administration of great tea is a potential strategy in treatment of prostate cancer, since great tea upregulates the expression of DAB2 to inhibit Akt and ERK1/2 signaling pathways [138]. It is held that the expression of miR-93 is higher in tumor cells compared to the normal cells making it an appropriate prognostic signature [140]. There a number of genes that are affected by miR-93 and TGFBR2, ITGB8 and LATS2 are among them. Accumulating data demonstrates that these three genes are responsible for proliferation and invasion of cancer cells [141–143]. As an oncogenesis miR, miR-93 elevates the proliferation, invasion and metastasis of prostate cancer cells by enhancing the expression of TGFBR2, ITGB8 and LATS2 [144]. Capicua (CIC) is a HMG box-containing transcriptional repressor that plays a significant role in preservation of homeostasis [145–147]. Interestingly, studies have revealed the potential role of CIC in pathogenesis of different cancers [148–150]. However, CIC reduces the proliferation and malignancy of prostate cancer cells. As an oncogenesis miR, miR-93 activates miR-106b/miR-375 to downregulate CIC-CRABP1 leading to the enhanced proliferation and migration of prostate cancer cells [151].

### Hepatocellular carcinoma

A growing body of evidence demonstrates that hepatocellular carcinoma cell (HCC) is a public health difficulty in both developing and developed countries [152, 153]. It is held that the deregulation of lncRNAs is responsible for generation of cancers, particularly HCC and contributes to their malignancy [154–158]. Besides, the resistance of HCC cells to chemotherapy reduces the efficiency of anti-tumor drugs. Expression investigation of lncRNA SNHG16 in both HCC cell lines and tissue revealed the minimal expression of SNHG16. Enhancing the expression of lncRNA SNHG16 demonstrated the reduced expression of miR-93 that in turn diminishes tumor growth in vivo and suppresses 5-fluorouracil (5-FU) resistant [159]. Multiple studies have shed some light on the impact of lncRNA LINC00472 in cancer malignancy. It seems that upregulation of this lncRNA diminishes the viability and proliferation of breast cancer cells and its low expression is associated with poor prognosis of patients with breast cancer [160–162]. Moreover, reduced expression of lncRNA LINC00472 enhances the migration and invasion of colorectal cancer cells [163]. It appears that miR-93-5p is a target of LINC00472. In order to suppress the malignancy of HCC cells, this lncRNA down-regulates the expression of miR-93-5p/PDCD4 axis to stimulate apoptotic cell death [164]. TP53INP1, CDKN1A and TIMP2 are able to regulate the proliferation and growth of tumor cells [165, 166]. It seems that exosomal miR-93 exerts stimulatory impact on the malignancy and invasion of HCC cells by down-regulation of TIMP2/TP53INP1/CDKN1A axis [167]. Peroxisome proliferator-activated receptor gamma coactivator-1 alpha (PPARGC1A) is suggested to be involved in generation of a number of disorders, particularly cancer [168, 169]. This factor contributes to the mitochondrial biogenesis and its expression undergoes inhibition in cancer cells [170, 171]. In respect to the anti-tumor activity of PPARGC1A, miR-93-5p suppresses its expression to enhance the proliferation and malignancy of HCC cells [172]. Programmed cell death 4 (PDCD4) induces programmed cell death to reduce the viability of cancer cells [173]. The expression of PDCD4 undergoes down-regulation in a number of cancers [174]. This protein remarkably diminishes the invasion of tumor cells by EMT modulation [175]. Notably, miR-93 inhibits PDCD4 by directly targeting its 3^/^-UTR resulting in enhanced migration of tumor cells via EMT stimulation [176, 177].

### Breast cancer

Breast cancer is one of the leading causes of women’s death with high metastasis capability [178, 179]. There are a number of molecular signaling pathways involved in regulation of biological processes such as cell proliferation and cell differentiation, and signal transducer and activator of transcription (STAT) is one of them [180, 181]. It has been revealed that STAT signaling pathway undergoes deregulation in a variety of disorders, particularly cancer [182, 183]. It seems that STAT3 is a target of miR-93 in breast cancer cells. LncRNA H19 inhibits the down-regulation of miR-93 to enhance the expression of STAT3 signaling pathway leading to the increased proliferation and metastasis of breast cancer cells [184]. Besides, the upregulation of exosomal miR-93 in ductal carcinoma in situ (DCIS) patients is a prognostic signature of breast tumors [185]. One of the troublesome problems faced in cancer therapy is the resistance of cancer cells to chemotherapeutic agents [186, 187]. Expression evaluation of miR-93 in breast cancer cell lines showed that miR-93 undergoes down-regulation in these tumor cells. Enhancing the expression of miR-93 is associated with high anti-tumor activity of chemotherapeutic agents by reducing the expression of Bcl-2 and P-glycoprotein (P-gp) [188]. Accumulating data demonstrates that miRs contribute to the stimulation of EMT in breast cancer [189–192]. Moreover, EMT mechanism induces drug resistance in cancer cells [193–196]. On the other hand, PTEN enhances the chance of drug resistance by EMT induction [197, 198]. MiR-93 mediates the resistance of breast cancer cells into doxorubicin (DOX) by EMT induction via targeting PTEN [199]. Another study puts emphasis on the downstream target of PTEN, so that during breast cancer progression, PTEN upregulation inhibits PI3K/Akt signaling pathway. However, miR-93 reduces the expression of PTEN to upregulate the expression of PI3K/Akt signaling pathway leading to the promotion of cell proliferation [200]. Previously, we mentioned that miR-93 induces drug resistance by activation of EMT mechanism. However, a study conducted by Xiang and colleagues provides controversial findings about the role of miR-93 in breast cancer. Based on the results of this study, miR-93-5p blocks both STAT3 and megakaryoblastic leukemia/myocardin-like 1 (MLK-1) as important regulators of cellular metabolism to suppress EMT resulting in reduced migration of breast cancer cells [201]. WNK lysine deficient protein kinase 1 (WNK1) is ubiquitously expressed in all tissues essential for embryogenesis [202–204]. WNK1 is capable of regulation of angiogenesis, cell proliferation and cell survival via targeting various pathways such as Smad/Tgfb, Erk5/MAPK and PI3K [205–207], demonstrating the potential role of WNK1 in tumorigenesis. By inhibition of WNK1, miR-93 diminishes the migratory capability and invasion potential of tumor cells [208].

### Gastric cancer

Gastric cancer [209] is a malignant cancer with high prevalence worldwide that has higher incidence rate in Asia and Western countries [210–213]. MiR-93 can function as a biomarker for GC diagnosis since its expression undergoes upregulation during the metastasis of GC cells into lymph node [214]. A variety of molecular pathways have been recognized as determining factors in GC progression. Hippo signaling pathway is suggested to be involvement in modulation of a number of physiological and pathological mechanisms including organ development, tissue regeneration and tumor suppression [215–217]. In respect to the role of Hippo signaling pathway in regulation of cell proliferation and apoptosis, this pathway exerts anti-tumor activity [218]. The effect of Hippo pathway dysregulation on the metastasis and invasiveness of tumor cells have been detected [219]. Epigenetic alterations are responsible for inhibition of MST1/2 and large tumor suppressors 2 (LATS1/2) as vital components of Hippo pathway during GC development [220]. Besides, protocadherin Fat4 (FAT4) is a main regulator of Hippo pathway that undergoes mutation in GC cells to ensure their proliferation and viability [221]. It is held that Hippo signaling pathway is one of the potential targets of miR-93-5p. Based on the oncogenesis effect of this miR on GC cells, it was found that during GC progression, upregulation occurs in the expression of miR-93-5p to suppress Hippo pathway via down-regulation of FAT4 and LATS2 leading to the enhanced migration and invasiveness of these malignant cells [222]. However, another study conducted by Meng and colleagues provides contrast results about the effect of miR-93 on Hippo signaling pathway. This study explains that miR-93-5p is able to promote the proliferation, migration and invasiveness of GC cells by stimulation of Hippo signaling pathway [223]. As it was mentioned, abnormal expression of JAK/STAT signaling pathway considerably elevates the malignancy of cancer cells [224, 225]. At the case of GC, a similar story occurs and miR-93 activates STAT3 signaling pathway to enhance GC metastasis [226]. However, it seems that this effect of miR-93 on STAT pathway is mediated by another target. Type I interferon (IFN1) has been reported to have anti-tumor activity [227]. IFN-1 reduces the activity of STAT3 by binding to the type-I interferon receptor 1 (IFNAR1) [228–230]. By inhibition of IFNAR1, miR-93-5p stimulates STAT3 signaling pathway to increase the malignancy and invasion of tumor cells [226]. On the other hand, lncRNA PTENP1 enhances the expression of PTEN (a tumor suppressor) to inhibit the proliferation and viability of GC cells. It seems that the stimulatory impact of lncRNA PTENP1 on PTEN is mediated by inhibition of miR-93 [231]. As it was mentioned, PDCD4 diminishes the viability and proliferation of tumor cells by stimulation of programmed cell death. The oncogenesis impact of miR-93 on GC cells is partially mediated through suppressing PDCD4 [232].

### Uterine cancer

Uterine cancer is one of the most common disorders of female reproductive system [233]. Obesity and bad lifestyles are the major risk factors of uterine cancer [234]. Based on the statistics of WHO, uterine cancer claims the fourth place among women’s cancer [152]. Abnormal expression of miR-93 occurs in patients with uterine cancer. These patients have a low expression of serum miR-93 and its expression has an intimate relationship with pathological staging and lymph node metastasis. High expression of miR-93 is associated with good prognosis and high survival rate of patients with uterine cancer [235].

### Adenoid cystic carcinoma

The primary lacrimal gland tumors have not high prevalence [236]. Adenoid cystic carcinoma (ACC) is suggested to be the most common form of malignant epithelial lacrimal gland tumors [237–239]. Radiotherapy and chemotherapy are the most frequent treatments of ACC [240, 241]. However, we have not witnessed a huge decrease in its eradication and recurrence. So, genetic manipulation is of interest in its treatment. The expression of miR-93 is higher in ACC tissues compared to the healthy ones. It is held that miR-93 upregulation enhances the metastasis of these tumor cells and stimulates epithelial-to-mesenchymal transition (EMT) via increasing the level of E-cadherin and reducing the level of N-cadherin [242]. Breast cancer metastasis suppressor 1 (BRMS1) has an intimate relationship with metastasis. Notably, BRMS1 exerts an inhibitory impact on the tumor cells by enhancing the expression of oncosuppresor miRs including miR-146a, -146b and -335 [243–246]. In the case of ACC, there is a reverse relationship between miR-93 and BRMS1L. Upregulation of miR-93 considerably diminishes the expression of BRMS1L to promote the malignancy and proliferation of cancer cells [242].

### Pancreatic cancer

Pancreatic ductal adenocarcinoma still has a high mortality and morbidity worldwide demanding novel strategies in its treatment [247–249]. Unfortunately, this kind of cancer is usually diagnosed at the advanced stages due to its special location and lack of sensitive approaches in its diagnosis [250]. Hence, studies should focus on treatment of this life threatening disorder at the end stages. In respect to the flexibility and complexity of cancer, targeting molecular pathway is the most important strategy. Runt-related transcription factor (RUNX) is a vital gene involved in progression of pancreatic cancer cells. Expression analysis shows the high expression of RUNX2 in pancreatic cancer tissues, while its expression is at the minimal level in healthy tissues [251]. Besides, it has been reported that RUNX3 contributes to the metastasis and migration of pancreatic cancer cells [252, 253]. A same story occurs for RUNX1, so that its expression undergoes upregulation in pancreatic tumor cells associated with poor prognosis and high malignancy of pancreatic tumor cells [254–257]. These studies highlight this fact that RUNX genes have a potential in cancer progression. Interestingly, it seems that RUNX1 exerts its stimulatory impact on the migration and malignancy of pancreatic cancer cells by inhibition of miR-93 since the overexpression of miR-93 diminishes the migration and invasiveness of pancreatic cancer cells [258].

### Renal carcinoma

Clear cell renal cell carcinoma (ccRCC) is the most frequent type of renal carcinoma affecting a high population of men and women around the world. Although surgery is a common option in treatment of ccRCC, it seems that patients demonstrate recurrence and metastasis after surgical resection with low survival rate [259–262]. This has resulted the willingness of scientists into other treatment options and it appears that anti-angiogenic agents are the most promising candidates. These agents can suppress the progression and malignancy of tumor cells with high capability [263–265]. Pigment epithelium derived factor (PEDF) has great anti-angiogenic activity beneficial in cancer therapy [266, 267]. It is held that miR-93-3p enhances the malignancy of ccRCC cells by stimulation of angiogenesis through down-regulation of PEDF [209]. Although it has been shown that TGF-β contributes to the induction of cell cycle arrest, accumulating data demonstrates that the resistance of cancer cells into TGF-β reverses its anti-tumor impact and this signaling pathway may enhance the progression of tumor cells [268–272]. The fundamental pathway involved in this function is various among different cancer types [273]. Mutations or deletions in Smad4 are responsible for promoting the progression of pancreatic cancer cells resistant to TGF-β [274]. However, TGF-β regulates an important axis in renal carcinoma cells that eventually reduces the proliferation of these malignant cells. It is held that TGF-β upregulation significantly diminishes the expression of miR-93 that in turn, stimulates RBL2 leading to the cell cycle arrest [275].

### Bladder cancer

Bladder carcinoma (BC) is supposed to be one of the malignant tumors of the urinary tract. In spite of the good prognosis of patients with BC, its incidence rate is still high [276]. Currently, Bacillus Calmette-Guerin is the best option for the treatment of BC [277]. Moreover, transurethral resectioning and chemotherapy are beneficial in BC therapy [278–280]. However, the resistance of BC cells remarkably reduces the potential of chemotherapy [281, 282]. Increasing evidence suggests that abnormal expression of miRs is an important factor in BC metastasis and progression [283, 284]. It seems that inhibition of miR-93 is advantageous in enhancing the anti-tumor activity of cisplatin (CS) against BC cells via stimulation of DNA damage [285]. LASS2 exerts inhibitory impact on BC cells and involves in chemo-sensitization [286]. It has been demonstrated that miR-93 induces the resistance of BC cells into chemotherapy by inhibition of LASS2 protein [285].

### Ovarian cancer

Ovarian cancer is a gynecological malignancy affecting a high population of women around the world with low 5-survival rate [287, 288]. Unfortunately, there are no advanced and sensitive tools for early diagnosis of ovarian cancer [289, 290]. So, treatment should be focused on using chemotherapeutic agents. In spite of the efficiency of chemotherapy, frequent application of these agents significantly reduces their capability in cancer therapy [291, 292]. As a consequence, scientists have had a special view into plant-derived natural products as agents with high anti-tumor activity that can be applied as adjuvant in cancer therapy [293–295]. Berberine (Brb) is a naturally occurring compound exclusively found in the members of *Berberis* family with high concentration in *Berberis vulgaris* [296]. This compound has a number of pharmacological activities such as antioxidant, anti-inflammatory, anti-diabetic and anti-tumor [297]. A combination of Brb and cisplatin significantly sensitizes ovarian cancer cells into apoptotic cell death and cell cycle arrest by inhibition of miR-93 and consequently, stimulation of PTEN/Akt axis [298]. MiR-93 down-regulation is a potent biomarker of ovarian cancer with the sensitivity as much as 93% [299].

### Colorectal cancer

Colorectal cancer (CRC) is considered as the third leading cause of death [300]. This life threatening disorder has high metastasis capability and there have been attempts to evaluate the molecular signaling pathways involved in its progression [301, 302]. It is held that lncRNA CA3-AS1 is capable of reducing the malignancy of colorectal cancer cells by inhibition of miR-93 and consequently, induction of PTEN as a tumor suppressor [303].

## MiR-93 and angiogenesis

Angiogenesis is one of the most important mechanisms involved in delivering oxygen and nutrients to the tumor cells [304]. It seems that this mechanism plays a remarkable role in a variety of stages of cancer such as proliferation and migration [305]. Anti-angiogenic agents have demonstrated great potential in inhibition of malignancy and invasion of cancer cells [306, 307]. There are two major problems associated with inhibition of angiogenesis: A) it appears that the introduced anti-angiogenic drugs are capable of suppressing angiogenesis in a just a number of cancers, and B) some of the tumor cells are able to advance without angiogenesis enhancing the complexity of cancer [308]. So, elucidating the molecular pathways involved in angiogenesis is suggested to be beneficial in cancer therapy. Upregulation of miR-93-5p increases the angiogenesis capability of human umbilical vein endothelial cells (HUVECs) leading to the improvement in blood vessel density, high proliferation and migration, and enhanced lumen formation and sprouting [309]. The interesting point of this study is the role of molecular signaling pathways. Epithelial protein lost in neoplasm (EPLIN) is a cytoskeleton-associated protein that plays a significant role in supervising the cell motility and actin dynamics. It has been demonstrated that high expression of EPLIN is related to the reduced ability of HUVECs in migration and tubule formation [310]. As a consequence, based on the efficiency of miR-93-5p in enhancing the angiogenesis and cell motility of HUVECs, it seems that this miR exerts inhibitory impact on EPLIN [309].

## Conclusion and remarks

**Fig. 1:**
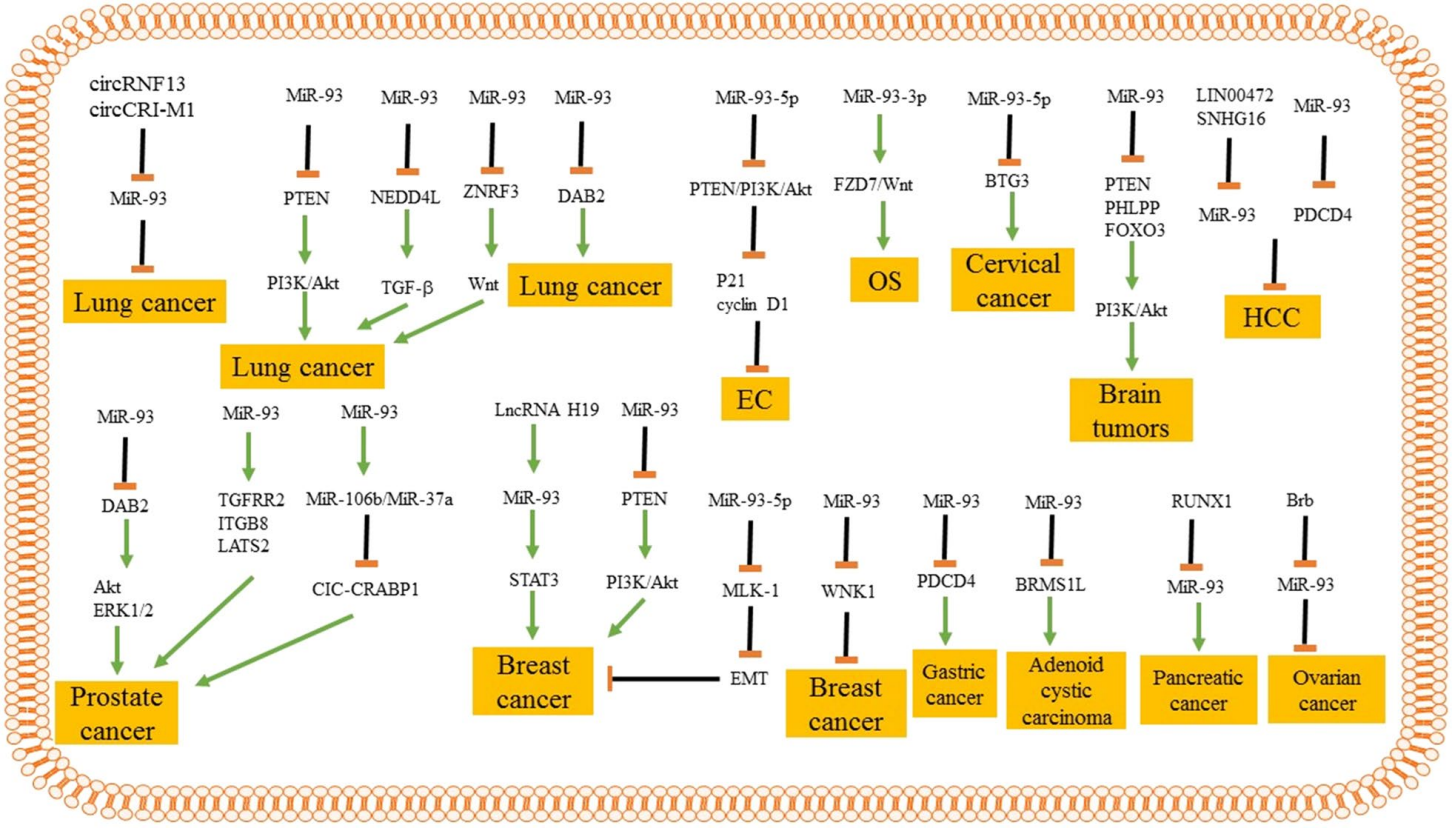
The involvement signaling pathways in the role of miR-93 in various cancers

This review provided a comprehensive discussion about the role of miR-93 in various cancer cell lines. Notably, all the studies conducted on the expression of miR-93 in lung cancer demonstrate that its upregulation is associated with poor prognosis of patients with lung cancer. More importantly, these studies imply that miR-93 is an oncogenesis miR in lung cancer that favors conditions into high proliferation and viability of lung cancer cells. The same story occurs in EC cancer. This miR not only serves as a biomarker during EC generation but also enhances the malignancy of cancer cells by inhibition of PTEN/PI3K/Akt signaling pathway. MiR-93/FZD7/Wnt axis is also important for promoting the progression of OS cells. It is noteworthy that the studies involving in the role of miR-93 in EC, OS and cervical cancers are low in number and more studies are required to clarify the oncogenesis or oncosuppressor impact of miR-93. But these experiments highlight the oncogenesis impact of miR-93. Interestingly, accumulating data demonstrates that miR-93 indirectly stimulates PI3K/Akt pathway to elevate the proliferation and malignancy of brain tumors. In this way, miR-93 suppresses the expression of PTEN, PHLPP, and FOXO3. It seems that miR-93 affects much more molecular pathways in prostate cancer. Figure 1 obviously shows these pathways. LncRNAs play a significant role in suppressing HCC. LncRNAs SNHG16 and LINC00472 exert inhibitory impact on the progression of HCC cells by down-regulation of miR-93. Investigating the role of miR-93 elucidated two major downstream targets known as STAT3 and WNK1. These two pathways are involved in regulation of a number of biological processes. MiR-93 suppresses WNK1 to reduce breast cancer malignancy, while it enhances the expression of STAT3. Figure 1 demonstrates the signaling pathways involved in the effect of miR-93 on various cancers. Besides, miR-93 is able to affect angiogenesis in cancer progression. MiR-93 reduces the expression of EPLIN to enhance angiogenesis. Finally, there is more studies that are supporting the role of miR-93 in cancer therapy [311–325], Table 1.

**Table 1:** The studies supporting the involvement of miR-93 in cancer malignancy

In vitro/in vivo	Effect on cancer	Major results	Refs.
Glioblastoma cells	Oncogenesis	MiR-93 enhances the malignancy and proliferation of glioblastoma cells by inhibition of autophagy via down-regulation of Beclin-1, ATG5, and ATG4B	[106]
EC cells	Oncogenesis	Reducing the capability of radiotherapy by inhibition of BTG3	[125]
HeLa and C‐33A cells cervical cancer tissues	Oncogenesis	LncRNA ZNF667 suppresses the invasion and malignancy of cancer cells by inhibition of miR-93-3p	[311]
Cisplatin-resistant A2780/DDP cell line	Oncogenesis	Inhibition of miR-93 by l-tetrahydropalmatine is beneficial in sensitizing of cancer cells to cisplatin-mediated apoptosis	[312]
Bladder cancer tissues and cells	Oncogenesis	Upregulation of miR-93 is related to the tumor stage and node stage via stimulation of PEDF	[313]
Cervical cancer and matched non-cancerous tissue samples	Oncogenesis	Overexpression of miR-93 and inhibition of CDKN1A is associated with poor prognosis of patients	[314]
Gastric cancer cells	Oncogenesis	By inhibition of TIMP2, miR-93 enhances the malignancy of gastric cancer cells	[315]
Primary colon cancer cells	Oncogenesis	By inhibition of miR-93, lncRNA LINC01567 reduces the proliferation an malignancy of cancer cells	[316]
Endometrial carcinoma tissues endometrial carcinoma cell lines HEC-1B and Ishikawa	Oncogenesis	Enhancing the proliferation and malignancy of tumor cells by stimulation of EMT	[317]
Ovarian carcinoma cell lines OVCAR3, SKOV3/DDP, and HO8910-PM	Oncogenesis	MiR-93-5p down-regulates the expression of RhoC to elevate the invasiveness of cancer cells	[318]
Human colon cancer tissue and colorectal carcinoma cell lines	Oncosuppressor	Diminishing the malignancy and migration of cancer cells by inhibition of Wnt signaling pathway	[319]
Hep-2 cells cancer tissues	Oncogenesis	MiR-93 binds to the 3^/^-UTR of cyclin G2 to inhibit its expression resulting in promoted proliferation of cancer cells	[320]
Breast cancer tissues	Oncogenesis	Overexpression of miR-93 occurs in triple negative breast cancer	[321]
Rat model of mammary carcinogenesis	Oncogenesis	Upregulated miR-93 suppresses the expression of Nrf2 to enhance the tumorigenesis of breast cancer cells	[322]
Tumoral and nontumoral colon tissues	Oncosuppressor	A decrease occurs in the expression of miR-93 in colon cancer cells	[323]
Early (recurrence within 12 months after surgery) and non-early relapse CRC patients CRC cells	Oncosuppressor	Reducing the progression and growth of cancer cells down-regulation of VEGF, p21 and ERBB2	[324]
Cisplatin-resistant ovarian cancer cells	Oncosuppressor	Upregulation of miR-93 reduces the expression of PTEN to stimulate Akt signaling pathway leading to the sensitization of cancer cells to chemotherapy	[325]

## Data Availability

The data sets supporting the results of this article are included within the article.

## Abbreviations

5-FU: 5-Fluorouracil
Ago: Argonaute
Brb: Berberine
BC: Bladder carcinoma
BRMS1: Breast cancer metastasis suppressor 1
circRNAs: Circular RNAs
CS: Cisplatin
ccRCC: Clear cell renal cell carcinoma
CRC: Colorectal cancer
DAB2: Disabled homolog 2
DAB2: Disabled homolog 2
DOX: Doxorubicin
DCIS: Ductal carcinoma in situ
EPLIN: Epithelial protein lost in neoplasm
EMT: Epithelial-to-mesenchymal transition
Nrf2: Erythroid 2-related factor 2
Exp5: Exportin-5
FOXO3: Forkhead box O3
GPR137C: G-protein coupled receptor 137C
HCC: Hepatocellular carcinoma cell
HUVECs: Human umbilical vein endothelial cells
LATS1/2: Large tumor suppressors 2
lncRNAs: Long non-coding RNAs
MLK-1: Megakaryoblastic leukemia/myocardin-like 1
miRs: MicroRNAs
miRISC: miR-induced silencing complex
MAPK2: Mitogen-activated protein kinase 2
NEDD4L: Neural precursor cell expressed developmentally downregulated gene 4-like
NDs: Neurological disorders
NSCLC: Non-small cell lung cancer
PPARGC1A: Peroxisome proliferator-activated receptor gamma coactivator-1 alpha
P-gp: P-glycoprotein
PHLPP: PH domain leucine rich repeat protein phosphatases
PEDF: Pigment epithelium derived factor
PCNSL: Primary central nervous system lymphoma
pri-miR: Primary-miR
PDCD1LG2: Programmed cell death 1 ligand 2
PDCD4: Programmed cell death 4
TRBP: RNA-binding protein
RUNX: Runt-related transcription factor
STAT3: Signal transducer and activator of transcription 3
TGF-β: Tumor growth factor-β
WNK1: WNK lysine deficient protein kinase 1
WHO: World Health Organization
ZNRF3: Zinc and ring finger 3
